# Ocins for Food Safety

**DOI:** 10.3389/fmicb.2019.01736

**Published:** 2019-08-02

**Authors:** Shilja Choyam, Alok Kumar Srivastava, Jae-Ho Shin, Rajagopal Kammara

**Affiliations:** ^1^Affiliated to AcSIR for Ph.D. Thesis, CSIR-CFTRI, Mysuru, India; ^2^Department of Protein Chemistry and Technology, CSIR-CFTRI, Mysuru, India; ^3^Food Safety & Analytical Quality Control Laboratory, CSIR-CFTRI, Mysuru, India; ^4^School of Applied Biosciences, Kyungpook National University, Daegu, South Korea

**Keywords:** bacteriocin, antagonism, biopreservation, dairy, meat, fruits, vegetables

## Abstract

The food industry produces highly perishable products. Food spoilage represents a severe problem for food manufacturers. Therefore, it is important to identify effective preservation solutions to prevent food spoilage. Ocins (e.g., bacteriocins, lactocins, and enterocins) are antibacterial proteins synthesized by bacteria that destroy or suppress the growth of related or unrelated bacterial strains. Ocins represent a promising strategy for food preservation, because of their antagonist effects toward food spoilage microorganisms, high potency, and low toxicity. Additionally, they can be bioengineered. The most common and commercially available ocins are nisin, plantaracin, sakacin P, and pediocin. Several ocins have been characterized and studied biochemically and genetically; however, their structure-function relationship, biosynthesis, and mechanism of action are not understood. This narrative review focuses primarily on ocins and their relevance to the food industry to help prevent food spoilage. In particular, the applications and limitations of ocins in the food industry are highlighted.

## Introduction

In humans, nutritionalΛ requirements are met by different food groups, e.g., dairy products, fruits, vegetables and legumes, meats, and grains, which are perishable and susceptible to microbial spoilage. Spoilage is characterized by changes in food quality that impact food appearance, color, texture, and/or flavor. Foods have a distinct and characteristic microflora from production to storage. Numerous microorganisms isolated from foods are capable of producing spoilage metabolites when unlimited growth is permitted (Gram, 1989). Food spoilage and waste represent an economic burden that cannot be controlled in spite of modern food technologies and preservation techniques. The typical microflora that develop during food storage have been extensively reviewed (Mossel et al., 1995). The main challenge is to ascertain the relationship between microbial colonies and microbial metabolites, the results of which would assist in the analysis and possible prediction of microbial spoilage (Borch and Agerhem, 1992; Drosinos and Board, 1995).

A vast number of spoilage microorganisms contribute to the deterioration of foods. However, the time between the appearance of large bacterial populations and the occurrence of spoilage events may vary depending on the food, intrinsic and extrinsic variables, and the growth characteristics of specific spoilage microorganisms (SSO). Therefore, identifying SSO and understanding the interactions between SSO and other microorganisms or their metabolites (i.e., synergism/antagonism) are important for estimating the potential damaging effects. Ultimately, microbial spoilage and the resulting (bio)chemical decomposition of foods should be evaluated.

Ocins are antimicrobial peptides or proteins produced by bacterial species and strains (Jack et al., 1995). Currently, ocins are used to control microbial growth in a wide variety of foods and beverages. Therefore, ocins have considerable commercial benefits. Even though thousands of ocins exist, most do not have appreciable activity essential for commercialization. The application of ocins in the food industry includes *ex situ* and *in situ* production by ocinogenic strains. The major obstacles of ocin use in the food industry are establishing large-scale production methods and implementing pilot and industry level applications. A standard method is the preparation of incompletely purified ocin concentrates as lyophilized powders. For example, nisin, which is produced by *Lactococcus lactis* fermentation of milk-based substrates and pediocin PA-1/AcH (pediocin PA-1) from *Pediococcus acidilactici*, which is commercialized as ALTA 2341 and used as a food protectant (Rodríguez et al., 2002). Even though nisin is extensively used in the food industry, it has a few limitations, including poor stability at high temperatures (Holcapkova et al., 2017), susceptibility to physiological enzymes (e.g., proteinase K, trypsin, and chymotrypsin), and effective activity at a very narrow pH range. Therefore, it is important to develop novel protein-based antimicrobials for applications in the food industry. Chikindas et al. (1995) reported the applications of bacteriocins, and Yang et al. (2014) discussed the antimicrobial activities of bacteriocins and their applications in foods and pharmaceuticals. The authors argued that several purified bacteriocins have been used in the food industry to extend food preservation time and treat certain diseases. Bacteriocins play significant roles in ecological homeostasis wherein they maintain the population dynamics within the species (Margaret and David, 1999). This function has been exploited for human betterment. Even though there are few review articles focused on bacteriocin databases, there are no studies on the enhanced production of recombinant bacteriocins. Consequently, we are unparalleled in this respect.

## Bacteriocins and Biofilms

Biofilms represent a microbial defense mechanism against antimicrobials. Pathogenic and infectious microorganisms such as methicillin-resistant *Staphylococcus aureus* (MRSA), *Pseudomonas aeruginosa*, *Gardnerella vaginalis, Staphylococcus epidermidis*, and *Streptococcus mutans* have the capacity to form biofilms. Referable to the lack of efficient, rapid and effectively penetratable antibiotics and also the drug resistance to biofilm formation made the pathogen removal not just hard but also impossible to wipe out completely. Small molecular weight bacteriocins represent a viable solution. Due to their low molecular weight, these bacteriocins have the ability to rapidly diffuse through biofilms and inhibit their formation. In the healthcare industry, biofilms represent a significant problem. Once the biofilm is formed in healthcare devices or instruments, the amount of antibiotic required for the eradication of the biofilm increases from 100 to 1,000×. Biofilm formation begins with the adherence of bacteria to a substrate, followed by maturation of the biofilm and subsequent release of clusters of cells from the matrix of the biofilm (Garrett et al., 2008; Flemming and Wingender, 2010).

It is challenging to dislodge mature biofilms; therefore, researchers have been evaluating ways to target the initial phases of biofilm formation (Dosler and Karaaslan, 2014). Unfortunately, the results have not been satisfactory. The current treatment consists on using antibiotics, antimicrobials, essential oils, and quorum sensing inhibitors. Nanoparticles may play an effective role as biofilm inhibitors (Algburi et al., 2017). Bacteriocins that have been used in the suppression of biofilm formation or growth include nisin, nukacin (Sashihara et al., 2000; Okuda et al., 2013; Chopra et al., 2015; Duarte et al., 2017), lacticin Q, sonorensin, and hyicin 4244. Nisin, which has a rapid penetration capacity and effective permeating activity, is an effective antibiofilm agent that can be bioengineered. Bioengineered or mutated forms of nisin have enhanced stable diffusibility (Davison et al., 2010; Field et al., 2015a,b,c).

The use of ocins in the food industry depends on their antimicrobial spectrum and the feasibility to introduce them directly into the food as a powder or indirectly via the inoculation of the food with an ocin-producing strain (Michael et al., 2018). Finally, functional aspects will be known by the use of the fermented product that has been produced in the presence of lactic acid bacteria. The antimicrobial activity of ocins may be estimated by disk diffusion, Well diffusion and also Unique well diffusion assay (Holder and Boyce, 1994; Kimara et al., 1998; Shilja et al., 2015). Both tests are highly dependent on the diffusion of ocins. Therefore, lack of diffusion may produce misleading results. The standard method for measuring ocin antimicrobial activity (Shilja et al., 2015) relies on a novel well-based assay that does not depend entirely on the diffusion of ocins. Several studies on the application of ocins in foods have been conducted. While most studies have focused on the use of ocins in the preservation of meat and dairy products, ocins may have potential in other food items, such as fruits and vegetables.

## Mechanism and Action of Nisin. Why Is Nisin Preferred?

Nisin production requires low cost ingredients; however, selling costs rapidly increase with commercially available bacterial ingredients.

## Social Acceptance

Currently, Nisin is not considered to be a market constraint in Europe or United States. Once acceptance has no issue in developed countries that do not cause product failure around the globe. Nisin has been extensively used for food safety and stability. The use of nisin represents a non-chemical, non-transforming approach that does not affect flavor, texture, or product structure.

## Nisin Mechanism and Action

There are different types of nisin molecules, e.g., nisin A, nisin Q, and nisin Z. These molecules mainly differ in their amino acid sequence; however, they possess similar antimicrobial action. Nisin Z, which is present as a monomer and dimer, has higher diffusibility compared with nisin A. Nisin A is a class I antimicrobial protein that displays a unique pore-forming activity. The pore forming activity is rapidly enhanced in the presence of a lipid II moiety (Breukink et al., 1999; Wiedemann et al., 2001). Nisin binds to the lipid II moiety through the N-terminus of two lanthionine rings, leading to the formation of a pyrophosphate cage around the head-group of lipid II (Van Heusden et al., 2002; Hsu et al., 2004; Wiedemann et al., 2004). Additionally, nisin inhibits cell wall biosynthesis by inhibiting PG synthesis. Segregation and loss of the lipid II moiety is another bactericidal mechanism of nisin. Research on nisin began in 2003 by Okeley, who characterized the components of the nisin biosynthetic pathway (Okeley et al., 2003). A few studies were performed during the 19th century. During the process of understanding its mechanism of action, several secondary nisin derivatives have been isolated. Nisin O was discovered by Hatziioanou et al. (2017). Thurusin CD is an ocin isolated from *Bacillus thuringiensis*. The mechanistic aspects of ocins clearly envisage that nisin and common antibiotics, such as vancomycin and metronidazole, have comparable activity (Rea et al., 2010).

## Advantages of the Review Paper ‘Ocins for Food Safety’ Over Other Reviews

The present manuscript comprehensively reviews the application of bacteriocins from all sources for the inhibition or elimination of food spoilage microbes for food safety. For easier understanding, the food sector was categorized, and applications of bacteriocins were mentioned separately. The successful application of native and recombinant bacteriocins against the food spoilage microbes was covered in the review. A follow-up article (Yang et al., 2014) related to the application of bacteriocins in foods and pharmaceuticals provided some information regarding the classification, mechanism of action, and general applicability against harmful microbes, but little information was provided about its specific application in different food categories. Another related review (by Chen and Hoover, 2003) mainly discussed the classification, genetics and mode of action of bacteriocins. The main aim of the present review/paper was to bridge the gap between the food industry and bacteriocin utilization and tells about how specifically one can utilize bacteriocins to the specific food commodity.

## The Bacteriocinogenics

A few bacteriocinogenics are used as cocultures that grow rapidly without interfering with the growth of the starter cultures. The bacteriocinogenics produce antimicrobials/ocins when required and are autonomous entities; therefore, their presence does not negatively affect the growth. Most or all of the probiotic microorganisms are considered bacteriocinogenic. Their presence is advantageous and useful in the production of safe foods.

## Applications of Ocins in the Dairy Industry

In 2015, the global dairy industry had an estimated milk production of 818 million tons (milk production in India during 2017 and 2018 was 176 million tons), of which 640 million tons corresponded to bovine milk. The leading producers of milk are Asia (29%), certain European countries (79.9%), North and Central America (18%), South America (10%), other European countries (9%), Africa (5%), and Oceania (5%; Gilles and Nico van, 2016; Bulletin of the International Dairy Federation 485/2016 ISSN 0250-5118). A vast array of products are made from milk, including raw, pasteurized, and dried milk, cream, butter, cultured buttermilk, paneer, sour cream, cottage cheese, yogurt, yogurt-based drinks and other fermented dairy foods, cream cheese, processed cheese, fresh soft cheeses, and ripened cheeses.

In the dairy industry, the pathogenic bacteria of primary concern include those that can live and multiply quickly in milk (even at 4°C) and different types of cheese, e.g., *Listeria monocytogenes*, *Staphylococcus aureus*, *Escherichia coli*, and *Salmonella* spp. (De Buyser et al., 2001). The decomposition of semi-soft and hard cheeses due to gas formation by *Clostridium tyrobutericum* has been responsible for substantial economic losses (Bergère and Lenoir, 2000). Nisin has been extensively tested in dairy products. For example, nisin has been used to inhibit gas production by *C. tyrobutyricum* in cheese (Hirsch et al., 1951; De Vuyst and Vandamme, 1994) and to inhibit *Bacillus cereus* growth in thermally treated cream during storage (at 5 IU g^-1^ nisin; Nissen et al., 2001). Nisin-coated polyethylene/polyamide packaging decreased the population of lactic acid bacteria (LAB), *Listeria innocua*, and *S. aureus* in packaged sliced cheese (Scannell et al., 2000). Lactococcal ocin, Lacticin 3147, was reported to have potential applications in dairy food preservation (Ross et al., 1999). Infant formulas containing lacticin 3147 suppressed the growth of *L. monocytogenes* and reduced *S. aureus* cell counts (Morgan et al., 1999). The viable cell counts of *L. monocytogenes* were reduced by 85% in yogurt and by 99% in cottage cheese within 2 h of lacticin 3147 powder addition (Morgan et al., 2001).

Parente and Hill (1992) concluced that ocin from *E. faecium* DPC1146 had a rapid bactericidal effect on *L. monocytogenes* in whole milk. Enterocin CCM 4231 reduced the active colonies of *S. aureus* SA1 in skim milk and yogurt (Lauková et al., 1999a,b). When added to goat cheese, concentrated enterocin CRL35 decreased *L. monocytogenes* population without affecting cheese quality (Farías et al., 1999). Similarly, pediocin PA-1/AcH decreased *L. monocytogenes* in several dairy products, including dressed cottage cheese, half-and-half, and cheese sauce (Pucci et al., 1988). A *Propionibacterium* P127 bacteriocin, propionicin PLG-1, inhibited several psychrotrophic spoilage and pathogenic bacteria (*Listeria monocytogenes*, *P. fluorescens*, *Vibrio parahaemolyticus*, *Yersinia enterocolitica*, and *Corynebacterium* sp.), suggesting a possible role as an antimicrobial food preservative (Lyon et al., 1993). Variacin, an ocin synthesized by *Kocuria varians* in a milk-based ingredient, hindered the growth and proliferation of *B. cereus* when added to chilled milk products, and vanilla and chocolate desserts (O’Mahony et al., 2001).

A natural variant nisin Z from *L. lactis* spp., lactis IPLA 729, which was identified in raw-milk cheese, reduced the growth of *C. tyrobutyricum* CECT 4011 (Rilla et al., 2003). Several studies (Ryan et al., 1996, 2001; Fenelon et al., 1999) have confirmed that the use of lacticin 3147-producing starters enhance cheese quality by inhibiting adventitious non-starter LAB flora during ripening. The transconjugants of lacticin 3147 effectively inhibited *L. monocytogenes* in cottage cheese (McAuliffe et al., 1999), mold-ripened cheese (Ross et al., 2000), and smear-ripened cheese (O’Sullivan et al., 2006). Enterocin A production resulted from a genetically modified strain of *L. lactis*. The starter was effectively utilized to inhibit *L. monocytogenes* populations in cottage cheese during the fermentation process (Liu et al., 2008). The bacteriocin of *E. faecium* 7C5 (Torri Tarelli et al., 1994; Folli et al., 2003) was stable for 40 days during ripening (Giraffa, 1995). The enterocin 226NWC, produced by *E. faecalis* 226, was active against *L. monocytogenes* during co-cultivation in skim milk at 30°C (Villani et al., 1993). *E. faecium* CCM 4231 synthesized a bacteriocin in Saint-Paulin cheese (Lauková et al., 2001). Furthermore, *E. faecium* DPC 1146 produced enterocin 1146 (enterocin A), and *E. faecium* strain RZS C5 produced an anti-listerial bacteriocin in cheddar cheese cocultures (Foulquié Moreno et al., 2003; Leroy et al., 2003).

Thermophilin 110, produced by *Streptococcus thermophilus* strain ST110, is a bacteriocin that inhibits the growth of Pediococcus, a food spoilage microorganism (Gilbreth and Somkuti, 2005). The food-grade lantibiotic Macedocin synthesized by *Streptococcus macedonicus* ACA-DC 198 was isolated from Greek Kasseri cheese and skim milk cultures with a nitrogen supplement (Tsakalidou et al., 1998; Georgalaki et al., 2002). *L. lactis* CL1 and CL2 produce pediocin PA-1 and derivatives (next generation molecules) that reduce *L. monocytogenes*, *S. aureus*, and *E. coli O157:H7* populations during fermentation (Rodríguez et al., 2005). The most important spoilage organisms and antagonistic bacteriocins in milk and dairy products are summarized in Table 1.

**Table 1:** Bacteriocins against milk spoilage microbes and pathogens.

Products	Spoilage microbes	Bacteriocins
Milk and milk products	*Listeria monocytogenes* *Staphylococcus aureus* *Salmonella* spp. *Escherichia coli* *Clostridium tyrobutyricum* *Clostridium botulinum*, *Bacillus cereus* *P. fluorescens* *Vibrio parahaemolyticus* *Yersinia enterocolitica* *Corynebacterium* sp. *Pediococcus* spp.	Nisin Z, Lacticin 3147, Enterocin CRL35, Pediocin PA-1/AcH, propionicin PLG1 Enterocin A, Enterocin 226NWC Enterocin CCM 4231, Enterocin AS-48 — Pediocin PA-1 Nisin Z, Thermophilin Nisin Nisin, Enterocin AS-48, Variacin Propionicin PLG-1 Propionicin PLG-1 Propionicin PLG-1 Propionicin PLG-1 Thermophilin 110

## Applications of Ocins in Meat and Meat Products

Meat and meat products are rich in proteins and provide a favorable environment for the proliferation of a variety of spoilage microorganisms. The availability of oxygen in meat at refrigeration temperatures allows the growth of Gram-negative aerobic bacteria, particularly *Pseudomonas*, and of *Carnobacterium*, *Lactobacilli*, and *Leuconostoc*, which predominate in anaerobic culture conditions (Borch et al., 1996; Gram and Dolgaard, 2002). Certain conditions in cooked meat products, such as low salt content (∼2%), a pH value of approximately 6.0, a water activity value higher than 0.945, and the absence of competing microbiota, create an ideal environment for foodborne pathogens and food spoilage microorganisms. Additionally, changes in the environmental gas composition of stored meat and meat products facilitate the growth of CO_2_-tolerant slow-growing bacteria, such as *Lactobacilli* (e.g., *Lactobacillus sakei* and *Lactobacillus curvatus), Leuconostoc carnosum*, *Leuconostoc gasicomitatum*, *Leuconostoc mesenteroides*, *Weissella* spp., and *Carnobacterium* spp. These spoilage bacteria contribute to sour taste and off-flavors, discoloration, gas, slime, and pH reduction (Aznar and Chenoll, 2006; Chenoll et al., 2007). Furthermore, *L. monocytogenes* growth and proliferation may be enhanced by meat processing conditions (Thévenot et al., 2006). Pediocin PA-1 retards the growth of Gram-positive meat spoilage microorganisms (Kalchayanand, 1990) and reduces *L. monocytogenes* populations (Nielsen et al., 1990) in beef. The recent studies have reported that pediocins have anti-listerial activity in meat (Motlagh et al., 1992; Taalat et al., 1993; Goff et al., 1996; Nieto-Lozano et al., 2006). The lactococcal bacteriocin lactocin 705 is useful in controlling *L. monocytogenes* in beef slurries (Vignolo et al., 1998) and in meat processing systems when combined with enterocin CRL35 and nisin (Vignolo et al., 2000). The addition of a freeze-dried piscicocin CS526 fermentate to ground meat reduced viable counts of *L. monocytogenes* (Azuma et al., 2007). Nisin in combination with lysozyme inhibited *B. thermosphacta* and LAB growth in vacuum-packed pork products (Nattress et al., 2001; Nattress and Baker, 2003). Various ocins, including pediocin AcH, sakacin P, carnobacteriocin from *Carnobacterium piscicola* L103, synthetic lactocin 705, and purified lactocin AL705 are effective antagonists of *B. thermosphacta* and Listeria in vacuum-packed meat products (Schlyter et al., 1993; Schöbitz et al., 1999; Katla et al., 2002; Castellano and Vignolo, 2006). Nisin was effective in preventing spoilage of bologna-type sausages by LAB (Davies and Delves-Broughton, 1999; Davies et al., 1999). Nisin provided a concentration-dependent inhibition of *L. monocytogenes*-induced spoilage when added to sucuk, a Turkish fermented sausage (Hampikyan and Ugur, 2007). In an experimental meat sausage model, the viability of *L. monocytogenes* and *S. aureus* was inhibited with enterocin AS-48 (Ananou et al., 2005a,b). When used as a starter for sausage fermentation, a sakacin P-producing *L. sakei* strain reduced counts in total bacteria, fecal *Enterococci*, and Listeria (Urso et al., 2006). Lacticin 3147, generated by a transconjugant of *L. lactis* DPC4275, significantly decreased *L. innocua* and *S. aureus* levels in beaker sausage (Scannell et al., 2001). The potential applications of bacteriocins in the meat processing industry are summarized in Table 2.

**Table 2:** Bacteriocins against meat spoilage microbes and pathogens.

Products	Spoilage microbes	Bacteriocins
Meat and meat products	*Pseudomonas* *Carnobacterium* *Lactobacillus* *Leuconostoc* *S. aureus* *Enterococci* *Brochothrix thermosphacta* *Listeria monocytogenes* *L. innocua*	— — Nisin Nisin Enterocin AS-48, Lacticin3147 Sakacin-P Nisin, Lactocin AL705 Pediocin AcH, Sakacin P, Carnobacteriocin, Lactocin 705 Pediocin PA-1, Piscicolin CS526, Lactocin AL705 Enterocin CRL35, Nisin, Enterocin AS-48, Sakacin-PLacticin 3147

## Applications of Ocins in the Poultry Industry

The poultry industry is negatively impacted by pathogens that contribute to significant product damage and serious economic losses. The major bacterial infections in the poultry industry are caused by *Vibrio cholera*, *Salmonella*, and *Clostridium*. Until now, the poultry industry has relied on the exploitation of various antibiotics for the treatment of bacterial infections. However, due to the emergence of antibiotic-resistant microorganisms and the import of antibiotic-contaminated poultry products, it is important to develop suitable and efficient antibiotic alternatives. On October 25th of 2018, a legislation, which will be implemented in 2022, was approved by the European Parliament to ban the prophylactic use of antibiotics in farming. Following the ban of all animal growth-promoting antibiotics by Sweden in 1986, the European Union banned avoparcin in 1997 and bacitracin, spiramycin, tylosin, and virginiamycin in 1999. This is not only imminent to ban the prophylactic use of antibiotics, but already partially applied the prohibition in the EU for example. Protein-based antibiotic molecules, such as nisin, may be considered suitable alternatives, because they have been tested and proven effective. Nisin addition to pasteurized liquid whole eggs reduced active *L. monocytogenes* populations, thereby increasing the shelf-life of the product under refrigeration conditions and preventing the proliferation of *L. monocytogenes* and *B. cereus* (Delves-Broughton et al., 1992; Knight et al., 1999; Schuman and Sheldon, 2003). Pediocin Pa1/Ach and nisin are antagonistic toward *L. monocytogenes* and act synergistically under heat treatment (Muriana, 1996; Knight et al., 1999). Nielsen et al., 1990 increased the thermal sensitivity of *Salmonella enteritidis* PT4 during pasteurization of liquid whole eggs and egg whites (Boziaris et al., 1998). Nisin coupled to high-pressure treatment of liquid whole eggs significantly reduced the viable counts of *E. coli* and *L. innocua*; these microorganisms were completely denatured after a month of refrigeration (Ponce et al., 1998). Holcapkova et al. (2017) concluded that nisin loses approximately 80% of its activity between 60°C and 70°C. The effects of ocins on spoilage microorganisms in the egg product industry are summarized in Table 3.

**Table 3:** Bacteriocins against egg spoilage microbes and pathogens.

Products	Spoilage/Pathogenic Microbes	Bacteriocins
Egg and egg products	*L. monocytogenes* *B. cereus* *Salmonella enteritidis* *L. innocua* *E. coli*	Nisin, Pediocin Pa1/Ach Nisin Nisin, Pediocin Pa1/Ach Nisin Nisin

## Applications of Ocins in the Aquaculture Industry

There are two different types of infections observed in aquaculture products, one occurs during the larval stage and the other occurs during storage. The aquaculture industry mainly cultures crabs, fish, and shrimp. *Aeromonas hydrophila*, *Vibrio cholera*, and *Staphylococcus haemolyticus* are the most common pathogens. A variety of microbial groups predominate during seafood storage depending on the preservation conditions and the nature of the product. In general, fish product preservation is performed with sodium chloride addition, slight acidification, and cold storage in vacuum packages. Other predominant microflora are LAB, primarily *Lactobacillus*, *Carnobacterium*, and some Gram-negative bacteria (e.g., *Photobacterium phosphoreum* and psychrotrophic *Enterobacteriaceae*). *L. sakei*, *B. thermosphacta*, *Serratia liquefaciens*, and *P. phosphoreum* were the only microorganisms that contribute to off-odors in cold-smoked salmon (CSS) samples (Stohr et al., 2001). The addition of acid or preservatives allow the growth and proliferation of lactobacilli and yeasts in some products, whereas mild heat treatment allows the propagation of surviving endospores of bacteria, such as *Clostridium* or *Bacillus* (Lindström et al., 2006).

Early studies have shown that nisin was not a suitable antagonist of *L. monocytogenes* in CSS because it only inhibited (i.e., was bacteriostatic, but not bactericidal) the growth in vacuum-packed fish products (Nilsson et al., 1997). However, nisin coupled with radio-frequency (RF) heating synergistically destroyed *L. innocua* and all mesophilic microorganisms. Populations of *L. innocua* in sturgeon caviar or ikura were killed at 65°C with RF-nisin treatment (Al-Holy et al., 2004). Nisin combined with heat or antimicrobial compounds inhibited *L. monocytogenes* and total mesophiles in sturgeon caviar (Al-Holy et al., 2005). Nisin Z, carnocin UI49, and bavaricin A, either in purified or crude form, have been evaluated for their ability to enhance the shelf-life of brined shrimp (Einarsson and Lauzon, 1995). The inoculation of CSS with cultures of *L. sakei* L6790, a sakacin P-producing strain, had a bacteriostatic effect on *L. monocytogenes*, similar to the effects of an isogenic *L. sakei* strain. Incomplete inactivation of *L. monocytogenes* was achieved when a sub-lethal concentration of purified sakacin P was combined with a bacteriocinogenic culture (Katla et al., 2001). The bacteriocins antagonistic to spoilage microorganisms present in seafood are summarized in Table 4.

**Table 4:** Bacteriocins against sea food spoilage microbes and pathogens.

Products	Spoilage/Pathogenic Microbes	Bacteriocins
Fish and other sea foods	*Lactobacillus* *Carnobacterium* *Photobacterium phosphoreum* Psychrotrophic *Enterobacteriaceae* *Clostridium* *L. monocytogenes* *L. innocua*	Nisin — Nisin — Nisin Nisin, Sakacin P Nisin

## Applications of Ocins in Fruits, Sprouts, and Vegetable Industry

This category of food includes unprocessed fruits and vegetables, processed ready-to-eat vegetables, canned products, fermented vegetables, fruit juices, drinks, and beverages. Sprouts have been consumed for many centuries in Asia and for 30 years in other parts of the world (Rosas and Escartin, 2000). Sprouts, which are considered to be a health food, are sources of foodborne illnesses caused by Salmonella, *E. coli O157*, and *B. cereus* (Beuchat, 1996, 2002; Taormina et al., 1999). Fresh produce, including raw celery, tomatoes, and lettuce, has been implicated in several listeriosis outbreaks (Beuchat, 1996). One of the most significant applications of bacteriocins is via competitive exclusion, in which non-pathogenic microorganisms inhibit the growth and proliferation of pathogens during sprouting. There are several reports on the isolation and identification of natural microorganisms from fresh produce that secrete antimicrobial substances (Carlin et al., 1996; Buchanan and Bagi, 1999; Liao and Fett, 2001; Wilderdyke et al., 2004). Among these microorganisms, the most effective are LAB strains that inhibit the growth of pathogenic microorganisms in ready-to-eat vegetables (Vescovo et al., 1996). *Lactococci*, which produce nisin, reduced *L. monocytogenes* populations in bean sprouts during refrigeration (Cai et al., 1997). Mundticin-producing strains of *Enterococcus mundtii* isolated from minimally processed vegetables prevented the growth of *L. monocytogenes* in a sterile vegetable medium (Bennik et al., 1999). Mundticin was an effective preservative for mung bean sprouts stored in a modified atmosphere (Bennik et al., 1999). Erwinia carotovora subsp. carotovora is a highly effective spoilage microorganism that causes soft rot in vegetables and fruits. The use of bacteriocins produced *ex situ* may avoid problems associated with *in situ* ocin secretion in vegetable foods.

There are no reports on any harmful effects of ocins on vegetable cells or tissues. Consequently, ocins might be suitable for the prevention of spoilage in fruits and vegetables, when either used alone or in combination with sanitizers. González-Pérez et al. (2018) induced bacteriocins in LAB to prevent spoilage in fruits and vegetables. However, the regrowth of surviving spoilage organisms of treated foods during storage needs to be taken into account. Pediocin, when used alone or with organic acid, was more effective than nisin alone in reducing *L. monocytogenes* populations (Bari et al., 2005).

Enterocin AS-48 was highly antagonistic toward *S. aureus* and completely inactivated *L. monocytogenes* and *B. cereus* in lettuce juice (Grande et al., 2005b). Nisin inhibited the growth of *L. monocytogenes* in honeydew melon slices, and its activity was increased when combined with a phage (Leverentz et al., 2003). Nisin was not effective in controlling spoilage of fruit in yogurt (Penney et al., 2004). In canned mango pulp, both nisin and bovicin HC5 inhibited *C. tyrobutyricum* growth and subsequent gas production (de Carvalho et al., 2007). In canned and cooked vegetables, endospore-forming bacteria are the main source of contamination. Several studies have confirmed that bacteriocins may eliminate endospore growth and proliferation and enhance the efficacy of thermal treatments to eradicate endospores in canned and cooked vegetables. In canned vegetables, nisin inhibited spoilage by non-aciduric (*Bacillus stearothermophilus* and *Clostridium thermosaccharolyticum*) and aciduric (*Clostridium pasteurianum*, *Bacillus macerans, Bacillus coagulans*) endospore-forming microorganisms (Thomas et al., 2000).

Nisin addition during processing resulted in a complete inhibition of bacterial growth and a 30-day extension in the shelf-life of pasteurized and vacuum-packed mashed potatoes when incubated in the presence of *Clostridium sporogenes* and *C. tyrobutyricum* spores (Thomas et al., 2002). The spoilage of fruit juices and beverages by *Alicyclobacillus* has been prevented with nisin addition (Komitopoulou et al., 1999; Yamazaki et al., 2000). Enterocin AS-48 completely inactivated *Alicyclobacillus acidoterrestris* for 3 months after inoculation of several fruit juice samples (Grande et al., 2005a). Nisin and enterocin AS-48 inactivated *A. acidoterrestris* endospores (Komitopoulou et al., 1999; Yamazaki et al., 2000; Grande et al., 2005a). In addition, enterocin CCM4231 produced by the enterococcal strain CCM4231 completely inhibited the growth of *L. monocytogenes* in soy milk and significantly decreased the viable counts of *S. aureus* (Lauková and Czikková, 1999). Nisin restricted the proliferation of *B. stearothermophilus* in soy milk and of thermophilic *Clostridia* in coconut milk/water (Thomas et al., 2000).

Potential biological control agents in the brewing industry include novel ocins produced by bacteriocinogenic strains of *L. sakei* and *L. mesenteroides* isolated from malted barley (Vaughan et al., 2001). Thermophilin 110 is antagonistic toward pediococcal strains (Gilbreth and Somkuti, 2005). Pediocin N5p from *P. pentosaceus* is resistant to physicochemical conditions of vinification including pH, temperature, ethanol, and SO_2_ (Strasser de Saad et al., 1995). Pediocin PD-1 synthesized by *P. pentosaceus* in beer was more effective than nisin and plantaricin 423 in the removal of mature *O. oeni* biofilms from stainless steel surfaces of tanks containing Chardonnay must (Nel et al., 2002). *O. oeni* can be effectively controlled in wine with the addition of pediocin PD-1 (Bauer et al., 2003). The main bacteriocins against fruit and vegetable spoilage microorganisms are summarized in Table 5.

**Table 5:** Bacteriocins against fruit and vegetable spoilage microbes and pathogens.

Products	Spoilage/Pathogenic Microbes	Bacteriocins
Fruits and vegetable	*Salmonella* *E. coli* O157 *B. cereus* *L. monocytogenes* *Clostridium sporogenes* *Alicyclobacillus acidoterrestris* *Staphylococcus aureus* *C. tyrobutyricum* *Bacillus stearothermophilusClostridium thermosaccharolyticum* *Clostridium pasteurianum* *Bacillus macerans* *Bacillus coagulans* *B. stearothermophilus* *Pediococci* *O. oeni*	Nisin — Enterocin AS-48 Mundticin, Pediocin, Enterocin AS-48, Nisin, Enterocin CCM4231 Nisin Nisin, Enterocin AS-48 Enterocin AS-48, Enterocin CCM4231 Bovicin HC5, Nisin Nisin Nisin Nisin Nisin Nisin Nisin Thermophilin 110 Pediocin PD-1

## Applications of Ocins in Cereals, Pulses, and Legumes

LAB bacteriocins ensure the safety and quality of cereal- and legume-based fermented foods. *L. lactis* subsp. *lactis* IFO12007, which produces nisin, was isolated from miso and used as a starter culture in the fermentation of cooked rice and soybean extract-supplemented rice koji. The bacteriocin-producing strain was grown in cooked rice and synthesized sufficient nisin to hinder the growth of *B. subtilis* without negatively affecting the growth of *Aspergillus oryzae*, which is needed in koji fermentation (Kato et al., 2001). Bacterial strains that negatively affect the long-term storage of cereals and pulses are presented in Table 6.

**Table 6:** Bacteriocins against cereals and pulses spoilage microbes and pathogens.

Products	Spoilage microbe	Bacteriocins
Cereals and pulses	*B. subtilis*	Nisin

## Applications of Ocins in the Baking Industry

Rope spoilage is due to less acidification, and also the presence of large amounts of sugar, fat or fruit (Beuchat, 1997). Therefore, the rope spoilage of wheat bread is a major concern in the baking industry. The main microorganism responsible for rope spoilage is *B. subtilis*; however, *B. licheniformis*, *B. megaterium*, and *B. cereus* may also be involved. *B. subtilis* and *B. licheniformis* have been inhibited in bread by nisaplin nisaplin^R^ and nisin-producing LAB (Rosenquist and Hansen, 1998). The addition of nisin as a powder prevents food spoilage caused by Gram-positive bacteria. However, as nisin is thermolabile, it needs to be incorporated following exposure to high temperatures.

## Action of Ocins in the Presence of Food Preservatives, Antioxidants, and Ingredients

Food processing is both a technique and an art. Food processing techniques adopted by most modern industries help retain food flavors for long periods of time or until the product reaches the consumer. Therefore, food processing not only ensures that the product is free of harmful pathogens and contaminants, but assists in retaining flavors and colors. The most commonly used food additives and antioxidant additives include sodium chloride, sucrose, acetic acid, ascorbic acid, benzoic acid, sodium benzoate, and sodium sulfite. The food preservatives and additives and their percentage used in food products are summarized in Table 7. Future studies should investigate the stability and functionality of ocins in the presence and absence of these additives.

**Table 7:** Most common food preservatives, food additives and conventional ingredients and their percentage used in food.

Preservatives	Percentage used
Sodium chloride	2 and 4%
Sucrose	1%
Acetic acid	0.1 and 0.3%
Ascorbic acid	0.015 and 0.055%
Benzoic acid	0.05 and 0.1%
Sodium benzoate	0.05 and 0.1%
Sodium sulfite	0.05 and 0.25%
Sodium metabisulfite	0.05 and 0.25%
Potassium metabisulfite	0.05 and 0.25%
Sodium nitrate	0.02 and 0.05 %
Sorbic acid	0.025 and 0.1%
Tartaric acid	1%
Citric acid	0.02 and 0.035%
Calcium propionate	0.1 and 0.4%

## The Need for Heterologous Expression and Production of Recombinant Ocins

Currently, ocin production is achieved by isolating the compound from cultured ocin-producing bacterial strains. This approach relies on the microorganisms and specific conditions under which high ocin expression may be feasible. The current demand for ocins is very high. Therefore, it is important to explore innovative ways of ocin production, e.g., via genetically engineered microorganisms such as *E. coli* and *Lactococcus lactis*. Between these microorganisms, *E. coli* is preferred because its genetic system is understood and can be exploited for the expression of any gene of interest (Beatriz et al., 2018). Even though several different expression hosts have been identified, BL21 DE3 and the plasmid pET are optimum (Studier and Moffatt, 1986; Rosenberg et al., 1987; Studier et al., 1990).

Most ocins are ribosomally derived; therefore, they cannot be cloned and expressed with a specific promoter. To express a specific ocin, a basic machinery for its synthesis and secretion is required. It is crucial to develop plasmids that contain the secretory signal, permease, the immunity gene, and the structural gene as a cluster. The construct needs to be made so that any open reading frame (ORF) coding for ocin may be easily introduced into the cassette for expression. Ultimately, the whole cassette should be inserted and directed under different promoters according to the demand. The promoter may be selected based on the characteristics of the ocins. A few ocins are toxic in nature; however, as soon as expression begins, they might have toxic effects on the host, leading to death. Therefore, it is important to exercise caution when selecting the promoter. Mesa-Pereira et al. (2018) discussed the production of ocins in heterologous hosts.

## Limitations of Ocins

Even though bacteriocins may be used in the food industry to eradicate disease-causing agents, they have some limitations. Considering that only hyperactive antimicrobial-producing strains may be considered for academic and commercialization purposes, there is no focus toward probiotic microorganisms that synthesize and secrete minimal quantities of ocins. *Bacillus licheniformis*, a GRAS microorganism, causes rope spoilage in apple cider (Grande et al., 2006).

The efficacy of bacteriocins might be product-dependent, e.g., the effectiveness of enterocin AS-48 against *S. aureus* is reduced in carbonara sauce (Grande et al., 2007). Chen and Hoover (2003) reported that moderate antimicrobial effects and limited specificity (narrow spectra) are major limitations in the application of bacteriocins in the food industry. Additionally, some bacteria are resistant to bacteriocins, e.g., Listeria is resistant to nisin, piscicosin, and pediocin (Gravesen et al., 2002; Martinez et al., 2005; Hayashi, 2007). Certain ocins may be inactivated by food components, and ocins may adsorb to the surface of the food, leading to proteolytic degradation. Information on the inactivation of bacteriocins in foods has been reported by Jung et al. (1992) and Ghalfi et al. (2007). The practical and daily problems faced by using ocins in food industry (for food preservation) are inactivated by food components a major problem. It is almost impossible to find the exact cause of inhibition.

**FIGURE 1:**
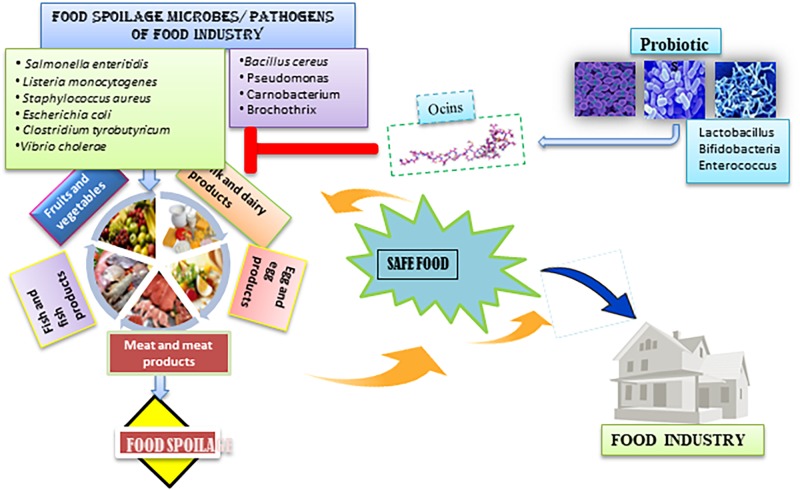
The various food spoilage microbes and pathogens that affect the different food industry. The influence of ocins to create safe food.

Collagen-like substances in food products or in supernatants of *Pichia pastoris* inhibited pediocin-PA1 bacteriocin activity (Beaulieu et al., 2005). Even though bacteriocins are not chemical-based compounds, they may alter food flavors and taste. It has been reported that the introduction of a few bacteriocins to foods results in loss of taste and in changes in odor (Drider et al., 2006). Regulatory concerns in developed countries hinder the applications of bacteriocins in the food industry. The various food spoilage microbes and pathogens that affect the different food industry and influence of ocins to create safe food has been depicted in the Figure 1.

## Conclusion and Future Perspectives

The applications of bacteriocins in food processing have been researched, with significant efforts devoted toward the preservation of meat and dairy products. Ocins may be highly effective in the preservation of fish, fruit, and vegetable products. Ocins, whether produced *ex situ* and added during processing or produced *in situ* by bacteriocinogenic strains, provide a natural antimicrobial barrier against pathogenic and spoilage bacteria. Several studies have focused on the introduction of bacteriocinogenic strains in foods. Compared with *ex situ* preparations, *in situ* ocin production offers several advantages. The scientific community is concerned about the use of chemicals and other food preservatives that might adversely affect human health. Ocins are natural compounds isolated from probiotic GRAS bacteria that are suitable preservatives in food and food products. Further studies are required to understand the mechanism of action of ocins. Modern biological techniques should be exploited to increase ocin production in heterologous hosts that will subsequently increase applications in the food industry. The ocin database developed by the Kammara group and available at ocins.cftri.com/ocins/ is the first database detailing food products, spoilage microorganisms, and corresponding bacteriocins. The information presented in the database might assist in the development of safe foods for the food industry (Choyam et al., 2019).

## Author Contributions

SC executed the idea. RK conceived, planned, designed the study, analyzed the data, interpreted the results, and wrote the manuscript. AS reviewed and prepared the final manuscript. J-HS involved in modifying the manuscript during the revision and contributed by introduction of data such as limitations, Bacteriocinogenics etc.

## Conflict of Interest Statement

The authors declare that the research was conducted in the absence of any commercial or financial relationships that could be construed as a potential conflict of interest.
